# Call for Physicians from the Sandwich Islands

**Published:** 1835

**Authors:** 


### XVI.—Call for Physicians from the Sandwich Islands.

We have lately received the following letter from one of the physicians attached to the Mission on these islands, under the direction of the American Foreign Missionary Society; and publish it for the information of such young physicians in the Valley of the Mississippi, as may, from religious feelings, have a disposition to connect themselves with remote missionary establishments.

*Sandwich Islands, Dec.* 19*th*, 1834.

Dr. Daniel Drake, Professor, etc. Cincinnati, Ohio:

Dear Sir, At a general meeting of this mission, held in June and July last, it was resolved, "That letters be addressed by us to the principal colleges and literary institutions in the United States, on subjects connected with the conversion of the world." From the commencement of our mission we held correspondence with societies and individuals in many of the colleges and theological schools, and have presented the command of Christ, "Go ye into all the world and preach the gospel to every creature," and have the importance of obedience, by exhibiting the appalling condition of 600,000,000 of our fellow beings, living in sin and without God in the world. Our appeals have hitherto been made to those preparing to preach the everlasting gospel, and we have called and invited them to come over and help us. But there is a field open, of wide extent, for the benevolent efforts of men of every profession. Particularly is there need of the labors of pious and well informed physicians.

Our mission at present consists of fifty-seven adult persons and sixty children, one hundred and seventeen persons in all, and a reinforcement of six or more is on their way out. The families are dispersed over five different islands, thro' an extent of 400 miles, and tho' there are three physicians devoted to their medical wants, the supply is found to be very inadequate. The frequency of the calls, and the irregular means of conveyance, from island to island, render it impossible for them to pay suitable and requisite attention. A physician could sooner, more conveniently and cheaply go from Boston to Charleston, S. C., or to Detroit, Michigan, than some of our requisite visits can be made, and at this time several families suffer for need of medical aid. They are so remotely situated that our physicians cannot attend to all. Two additional medical men are immediately needed, that there may be one on each principal island. Much suffering and anxiety might thus be prevented among those who have voluntarily forsaken all for the kingdom of Christ's sake.

Another extensive and important field of usefulness is open in the practice afforded among the natives. The multitudes of diseases of long standing and the frequency of deaths which might apparently be prevented by an enlightened treatment, are evidences of the insufficiency and imbecility of their medical practice, much of which consists in charms and incantations, and the whole is destitute of skill or correct principle. We have known individuals suffering with both acute and chronic diseases, the effect of drastic medicines taken, in perfect health, from a belief that they might soon be sick. And we have known others, moving about in the morning, without an appearance or a symptom of disease, at night the victims of the same absurd and superstitious practice. But we have lived here to see these customs decreasing, and a preference felt for the medicine of the foreigner, wherever it has been used.

As teachers in schools, or in imparting divine truths to this people, there are no limits to the field, and every moment that the missionary physician can spare from his profession, can be profitably employed. We hope ere long to see statesmen, and men eminent in every profession, among the natives of these islands. There are talent and genius, and powers, susceptible of being expanded to an unlimited extent, and we have evidence of the existence of minds of the highest order, among the people around us. We have a high school in successful operation, where we shall ere long have lectures on the different departments of science, but we need provisions for carrying the scholars through the different professions, and for this purpose need the men on the ground, acquiring the language, learning the ways and manners of the people, and in every way qualifying themselves for the work. Our cause, we feel, is urgent. It is no less than raising 130,000 souls from a state of degradation, or little superior to the brutes, and elevating them to the condition of enlightened and Christian countries. And are there not, dear sir, young men under your tutelage willing to assist in the work? Are there not those possessing piety, and benevolence, and self-denial enough to constrain them to forego the prospects of wealth and comfort, among an enlightened society, if they can possess the nobler, more satisfying prospect of alleviating the moral and spiritual condition of thousands ready to perish? Can you not, will you not, tell them of our wants, of the wants of this benighted land, and urge them to come to our aid?

We shall be happy, dear sir, to hear from you. Communications addressed to the Sandwich Island Mission, and sent to the Missionary Rooms, Boston, will be forwarded, and receive due attention from us. In behalf of the Sandwich Island Mission,

I am yours, etc.

ALONZO CHAPIN, M. D.

We cannot but hope, that some of our younger brethren will feel inclined to accept of the invitation here so eloquently given. Christian misssons are not now what they once were, mere projects of religious conversion, unaccompanied with preparatory and co-operative efforts at the introduction of learning and the necessary and simple arts of civilized life, for now the latter are, indeed, rather placed in advance; and among the whole of the efforts of Christendom in the uncivilized world, there is not one which seems to have been made more rigidly on this principle or with more striking succes than this, whether we refer to the introduction of knowledge or religion. Here then is a great object of benevolence, altogether worthy of the attention of the man of science, as well as the Christian, and why should not the medical profession contribute its aid? an aid which can be afforded from no other quarter. We cannot but think, that a residence, for a few years at least, in the Sandwich Islands, would be attended with many agreeable results to the young physician, who might, in his novitiate, go thither. The climate of these islands is temperate, pleasant, and salubrious, notwithstanding they lie within the tropics.

The study of their natural productions would be interesting, and the opportunities of becoming acquainted with the physical and moral temperament, the modes of living, and the peculiar diseases of a portion of the Malayan variety of the human race, would of course enlarge and enrich the mind of the observer, to say nothing of the valuable observations that might be made on the ocean, and the ports touched at in the voyages out, and home again, in the event of a return. Should any of our young readers embark in this enterprize, we shall hope ere long to present our readers with a chapter of interesting scientific details from OCEANICA.

